# Biosurfactant Production Using Mutant Strains of *Pseudomonas aeruginosa* and *Bacillus subtilis* from Agro-industrial Wastes

**DOI:** 10.34172/apb.2021.063

**Published:** 2020-10-20

**Authors:** Samson A. Adejumo, Angus Nnamdi Oli, Ebere Innocent Okoye, Calistus Dozie Nwakile, Chioma Miracle Ojiako, Ugochukwu Moses Okezie, Ifeanyi Justin Okeke, Chijioke M. Ofomata, Anthony A. Attama, Jude N. Okoyeh, Charles Okechukwu Esimone

**Affiliations:** ^1^Department of Pharmaceutical Microbiology and Biotechnology, Faculty of Pharmaceutical Sciences, Nnamdi Azikiwe University, Awka, Nigeria.; ^2^Department of Pharmaceutical Microbiology and Biotechnology, Faculty of Pharmaceutical Sciences, Federal University Oye Ekiti, Ekiti State, Nigeria; ^3^Department of Pharmaceutics and Pharmaceutical Technology, Faculty of Pharmaceutical Sciences, Nnamdi Azikiwe University, Awka, Nigeria.; ^4^Department of Clinical Pharmacy and Pharmacy Management, Faculty of Pharmaceutical Sciences, Agulu, Nnamdi Azikiwe University, Awka.; ^5^Department of Pharmaceutics, Faculty of Pharmaceutical Sciences, University of Nigeria, Nsukka, Enugu State, Nigeria.; ^6^Department of Biology and Clinical Laboratory Science, Division of Arts and Sciences, Neumann University, One Neumann Drive, Aston, PA 19014-1298, USA.

**Keywords:** Biosurfactant production, Bacillus subtilis, Pseudomonas aeruginosa, Microbial biotechnology, Palm oil wastes, Abattoir soil

## Abstract

***Purpose:*** Biosurfactants are applied in drug formulations to improve drug solubility and in some cases, treat diseases. This study is focused on generating, extracting, purifying and then characterizing biosurfactants from bacterial isolates of palm oil wastes and abattoir soil origins.

***Methods:*** Eight bacteria were isolated from the soil and sludge samples, out of which four (50%) were found to produce biosurfactants. *Bacillus subtilis* (37.5%) and *Pseudomonas aeruginosa* (50%) were isolated and identified from these samples using mineral salt medium, nutrient agar and Cetrimide agar. Mutant isolates of *B. subtilis* BS3 and *P. aeruginosa* PS2 were used to produce biosurfactants using mineral salt medium as enrichment medium and extraction was done using membrane filter.

***Results:*** The mutant strains *B. subtilis* BS3 and *P. aeruginosa* PS2 generated biosurfactants that displayed significant solubility and dissolution properties by enhancing the percentage solubility of piroxicam to 62.86 and 54.29% respectively, and achieved 51.71 and 48.71% dissolution of the drug in 0.1N HCl.

***Conclusion:*** From the results obtained, the produced biosurfactants could serve as a better alternative to conventional surfactants. Notably, the study indicated that the biosurfactant produced by mutant strain of *B. subtilis* produced more potent activities (surface tension reduction ability, high emulsification) than those of *P. aeruginosa*.

## Introduction

Surfactants are organic molecules containing hydrophilic and hydrophobic ends and are capable of lowering the tension co-existing between two phases that cannot mix.^1^ They are extensively used in several industrial processes to achieve several goals.^2^ Most of the surfactants in current use are produced from petroleum and/or other chemical products and are usually not eco-friendly. “These compounds are often toxic to the environment and their use may lead to significant environmental problems, particularly in cosmetics as these surfactants inevitably end up in the environment after use”.^3^ Eco-toxicity, bioaccumulation and non-biodegradability of most petro-chemical surfactants are seriously possing some global concerns.^4^

Biosurfactants, on the other hand, are substances produced by microorganisms and can lower the surface tensions and/or the force that exists at the point where two immiscible phases intercept. They resemble synthetic surfactants and are amphiphilic in nature but are produced by microorganisms during metabolic processes.^5,6^ They are usually safe and easily degraded in living tissues. They promote foaming, lower the surface tension and create stable emulsions.^7^ Biosurfactants such as surfactin, fengycin, iturin and bacillomycins act as wide-spectrum bioactive compounds with applications in both pharmaceutical and biotechnological industries.^8^

Presently, synthesized culture media used in biosurfactant generation heightens the cost of commercial biosurfactants. However, biosurfactants can be produced by fermentation from sugars and various cheap renewable sources particularly agro-industrial substrates.^9^ When most drugs are administered, they are firstly absorbed systematically and then transported to their site(s) of action. The two vital processes involved are: dissolution followed by absorption. Biosurfactants have intrinsic abilities to enhance drug dissolution and solubility of drugs by lowering melting point due to their ability to lower the interfacial tension as well as micellar formation.^10^

The present study looks at the pharmaceutical evaluation of biosurfactants produced by mutant strains of *Bacillus subtilis*and *Pseudomonas aeruginosa* obtained from abattoir and palm oil-contaminated soil.

## Materials and Methods

### Preparation of turbidity standard equivalent (0.5 McFarland)

A milliliter of conc. Sulphuric acid was added to 99 mL of water and then vortexed to thoroughly mix. A 0.5 g of anhydrous barium chloride (BaCl_2_) was dissolved in 50 mL of distilled water. A turbid suspension of 0.6 mL of BaCl_2_ in 99.4 mL of Sulphuric acid was then obtained by mixing. A 5 mL of the turbid suspension was added to a capped tube of same type as used for preparing the test and control inocula. The McFarland turbidity standard was stored in a well-sealed container in the dark at a temperature of 25°C.

### Induction of mutation on organisms using UV irradiation

*Bacillus subtilis*(BS3) and *Pseudomonas aeruginosa*(PS2), were isolated from abattoir and oil-contaminated soil and sludge samples. The isolates were purified through subculturing. Then the suspensions of *P. aeruginosa*(PS2) and *B. subtilis*(BS3)were prepared according to 0.5 McFarland turbidity standards.^11^ A 20 µL of suspension was inoculated and spread on to nutrient agar medium in Petri dishes and exposed to UV rays (from fluorescent lights in ESCO laminar flow with an emission spectrum of 250-370 nm) for 5, 10, 15, 30, 60, 120, 300, 600 and 900 seconds at a distance of 30 cm in all cases. After the exposure to UV light, all the samples were incubated at 37°C for 24 hours. Microscopic and biochemical tests were carried out on resulting colonies to be able to select mutant strains for subsequent fermentation stage. All results were recorded in triplicates. The isolates were maintained in separate nutrient agar plates with the following compositions (g/L): peptone-5.0; sodium chloride-5.0; Agar-15.0; yeast extract-2.0; beef extract-1.0 at pH of 7 in storage temperature of 4°C and sub-cultured once in 2 weeks.

### Fermentation media and cultivation conditions

The freshly prepared microbial mutants/inocula (5%) were obtained from the stock culture of each of the selected bacteria isolates, adjusted to 0.5 McFarland turbidity standard and then grown^12,13^ in a rotary shaker containing 150 mL of mineral salt medium [potassium dihydrogen phosphate (3 g/L), yeast extract (10 g/L), sodium nitrate (30 g/L), magnesium sulphate (3 g/L), ferrous sulphate (0.01g/L), calcium chloride heptachlorate (0.02 g/L), and glucose (40 g/L) as sole carbon source] as described by Ghribi and Ellouze-Chaabouni et al.^14^ The medium, adjusted with 1M HCl to a hydrogen ion index of 6.8, was first sterilized and trace element solution containing zinc sulphate (0.29 g/L); calcium chloride (0.24 g/L); copper sulphate (0.25 g/L) and manganese sulphate (0.17 g/L) was added. The culture temperature, agitation rate and duration of fermentation were 37°C, 200 rpm and 7 days respectively.

### Physical characterization of bacterial mutants

The morphology of the isolated organisms was studied by observing the heat fixed stains of isolates under ×100 objective lens of Olympus microscope. Some biochemical tests (Gram staining, oxidase test, catalase test, starch hydrolysis and spore staining) were carried out to characterize the microorganisms up to species level.

### Screening for biosurfactant producing organisms

The pure mutant cultures were screened for biosurfactant production as described below.

#### Oil displacement test

A 20 mL distilled water at neutral pH was placed in a 90 mm petri dish. Five milliliter of vegetable oil was added to the plate. A 500 µL of the culture solution was gently put on the center of the oil film. After 30 seconds, the diameter and area of oil displacement were visualized under visible light and then measured. The ability of the mutants to displace the vegetable oil was observed for 120 seconds.^15^

#### Blood hemolysis test

The use of blood hemolysis as a method of screening for biosurfactant production has been established, as biosurfactants can cause lysis of erythrocytes.^16^ Twenty-four hours fresh colonies from the mutants were inoculated on blood agar plates and incubated for 24 hours at 37°C. A clear zone around the colonies indicated the presence of biosurfactant producing organisms.^17^

#### Emulsification test

Fresh colonies of the mutant strains were suspended in 2 mL of water and vortexed vigorously with 2 mL of vegetable oil for 5 min. The mixture was left for 24 hours to settle with a resultant three layers: water layer, emulsion layer and oil layer. Water mixed with vegetable oil only was used as control. The emulsification activity was calculated using following equation.^18^

Eq. 1 $$\text{Emulsion index}\left({\text{E}}_{24}\right)=\frac{\text{Height of emulsion layer}}{\text{Total height of liquid column}}\times 100\text{\%}$$

#### Biosurfactant extraction and preliminary analysis

The 7 day-old fermentation broths were first subjected to 4000 ×g centrifugation for a period of 15 minutes and allowed to stand overnight at 4°C. Thereafter, the supernatant was further subjected to ultracentrifugation at 8000 ×g for 15 minutes.^15^ The crude biosurfactant was sterilized by ultrafiltration using a sterile 0.4 μm millipore membrane filter. The surface tension was determined using a Kruss Tensiometer (KSV Sigma 702) as described in literature.^19^ The surface tension measurement was carried out at 35 ± 2°C. First, the platinum ring of the tensiometer was immersed into the solution for 15 minutes to equilibrate and then slowly dragged along the air-liquid interface until a constant reading was obtained. The surface tension was measured in Dyne per centimeter (D/cm). After every measurement, the wire ring of the instrument was rinsed with distilled water and then flammed to sterilise. Instrument calibration was done at 71.5 D/cm ±0.5 being the surface tension of distilled water and at neutral pH before each set of experiments while Tween 80 was used as a positive control. The surface tension was then plotted against biosurfactant concentration to obtain the critical micelle concentration (CMC).

Oil displacement test and determination of the emulsification index were carried out as earlier described.

#### Antimicrobial activity of biosurfactants

Six bacteria (viz: *Staphylococcus aureus*, *Salmonella*spp, *Shigella*spp*, E. coli*, *Vibrio* spp and *Listeria* spp) and three fungi (*C. albicans*, *Aspergillus flavus* and *Aspergillus niger*) obtained from Pharmaceutical Microbiology Laboratory of Nnamdi Azikiwe University, Agulu were used for the antimicrobial screening.

#### Antibacterial activity

This was evaluated following previously reported method^20-22^ with some modifications. Broth cultures of test bacterial isolates adjusted to 0.5 McFarland Standard were streaked on the surface of Mueller Hinton agar plates with the aid of sterile cotton swabs. All the culture plates were allowed to solidify for about 5 minutes. The agar well was bored using a sterile cork borer (6 mm in diameter) and filled with various concentrations of the biosurfactants. Thereafter, 30 μL of purified biosurfactant at different concentrations: 1.02, 2.04, 6.12, 12.14 mg/L were dropped into the respective sterile bored holes on the agar plates. The plates were incubated at 37°C for 24 hours and the zones of inhibition measured. Gentamicin (10 μg) was used as the positive control and dimethyl sulfoxide (DMSO), the negative control. Triplicate determinations were made.

#### Antifungal activity

Liquid cultures adjusted to 0.5 McFarland Standard of the test fungal organismswere aseptically spread out on the surface of sabouraud dextrose agar plates having 6 mm holes bored with sterile cork borer. Thereafter, 30 μL of various concentrations of purified biosurfactants (1.02, 2.04, 6.12 and 12.14 mg/L) were dropped unto the sterile bored holes on the plates. The fungal culture was incubated at 27°C for 72 hours and the inhibition zones diameter measured. Clotrimazole (50 μg) was used as the positive control and DMSO, the negative control. The exercise was carried out in triplicates.

#### Dry weight measurement of biosurfactants

Sterile dry petri dish was weighed and 5 mL of supernatants of the biosurfactants were transferred into the dish and heated in an oven to 100°C for 30 minutes. The biosurfactants dry weight was calculated as follows.^23^

Eq. 2 Dry weight=Weight of Dried plate −Weight of Empty plate

### Determination of the pharmaceutical profiles of the poorly-soluble piroxicam

#### Preparation of piroxicam and biosurfactant solid dispersion

The method described in literature^24^ was followed with some modifications. A 2 g each of piroxicam was separately mixed with 3 mL and 6 mL of purified biosurfactants using mortar and pestle. The mixture was kept in a desiccator containing charged silica gel and allowed to dry at ambient conditions to a uniform weight. The resulting powder was packed in screw-capped amber coloured glass bottles.

#### Determination of wavelength of maximum absorption (λmax) of piroxicam

As reported earlier^25^ with some modifications, 50 mg of piroxicam powder was carefully weighed and dissolved in 10 mL of methanol contained in a 100 mL volumetric flask. This was diluted to 500 µg/mL using 100 mL distilled water (pH 7.0). A 1 ml was withdrawn and transferred to another 10 ml volumetric flask and the volume made up to 100 mL with distilled water to get a concentration of 50 µg/mL. The 50 µg/mL solution was scanned using Agilent Cary 60 UV-Vis Spectrophotometer (Santa Clara US) in the range of 200–400 nm.

#### Construction of calibration curve for piroxicam

This was according to literature^26^ with some modifications. The stocks, 0.1, 0.2, 0.4, 0.6, 0.8, 1.0, 1.2, 1.4, and 1.6 mL of solutions were separately transferred into 10 mL volumetric flasks and volumes made up to 10 ml with distilled water using sterile micropipette to obtain concentrations of 5, 10, 20, 30, 40, 50, 60, 70 and 80 µg/mL respectively. The absorbance of the solutions was measured at 358 nm (λmax) using Agilent Cary 60 UV-Vis Spectrophotometer (Santa Clara US). All the studies were conducted in triplicates and the mean values used to construct the Beer’s curve.

#### Characterization of biosurfactant-piroxicam solid dispersions

Modified methods from literatures^27-29^ were followed.

#### Fourier transforms infrared spectroscopy (FTIR)

A 1 mg quantity of sample (solid dispersion or pure piroxicam) was thoroughly mixed with potassium bromide (100g), pressed to translucent pellets for 30 s and then scanned spectrophotometrically using Agilent Cary 60 UV-Vis spectrophotometer (Santa Clara US) in the range of 650–4000/cm with a resolution of 1/cm. Agilent Cary 300 WinUV software was used to analyse the IR spectra.

#### Differential scanning calorimetric (DSC) analysis

A weighted 4.3 mg quantity of sample (solid dispersion) was placed in the sample pan of the Mettler Toledo DSC 822e (Columbus, Ohio, USA) instrument and heated constantly at 12°C/min (using alumina as the internal standard and under gaseous nitrogen) to 400°C. A control was set up but this time, the sample pan was void and all results were taken in triplicates. The procedure was later repeated using pure piroxicam

#### X-ray powder diffraction

The solid state of piroxicam and biosurfactant-piroxicam complex was assessed in an X-ray powder diffraction (XRPD)instrument (MiniFlex, Rigaku, USA). CuK radiation was employed to analyse the diffraction and at the following conditions: angular range = 5-70 °C; voltage = 35 kV; current = 20 mA; temperature = 25 °C and scan rate = 2°/min.

#### Solubility studies

This was done following the procedure from previous literature.^30^ A 20 mg of piroxicam in biosurfactant samples was weighed into vials containing 10 mL of distilled water at pH 7.0. The vials were stoppered and shaken thoroughly for 30 min. The samples were then placed in water bath shaker and shaking continued for 48 hours. The temperature of the bath was maintained at 37°C. After 48 hours, the contents were centrifuged to clarify the mixture and filtered using 0.45 μm Millipore membrane filter. A 0.1 mL of the filtrate was withdrawn and made up to 4 mL with distilled water. Using Agilent Cary 60 UV-Vis Spectrophotometer (Santa Clara US) and applying the calibration curve equation, the samples were analyzed for piroxicam content at 358nm absorbance reading.

#### Dissolution studies

*In vitro*dissolution studies of biosurfactants were performed according as reported previously in the literature with some modifications^31^ using the Hanson Research SR8-Plusdissolution apparatus (Canada). Amount of sample equivalent to 20 mg of piroxicam was added to 400 mL of 0.1 N HCl (pH 1.2) in the dissolution chamber at 37 ± 2°C. The paddle was operated at 50 rpm and aliquots of 5 mL were withdrawn with equal fresh medium replacement at the intervals of 5, 10, 20, 30, 40, 50 and 60 minutes using sterile syringe. The withdrawn samples were filtered (Millipore membrane filter) and analyzed using UV spectrophotometer (Jasco, V-530, Japan) at 358 nm.^30^ Readings were taken in triplicates and average values of the results recorded.

### Statistical analysis

The results were analyzed using ANOVA, chi-square, Pearson correlation and linear regression in SPSS 20.0 and Microsoft Excel 2010 at *P* value <0.001.

## Results and Discussion

### Isolation and identification of selected strains

A total of five different bacterial isolates were obtained from the soil and sludge samples. The isolates include *Bacillus subtilis, Pseudomonas aeruginosa, Citrobacter*sp*, Escherichia coli,*and *Enterobacter sp.* Table 1 shows the result of microscopy and biochemical identification of the isolates. In this study, samples of oil-contaminated soil and sludge were obtained from palm oil processing site in Nise, Anambra State, Nigeria, while similar samples contaminated with animal fats were collected from an abattoir in Awka, Anambra State, Nigeria. *B. subtilis*and *P. aeruginosa*were isolated and identified from the four samples. The isolated strains BS1 and BS3 produced a distinctive blue-black coloration when grown on starch agar. The bacteria strain which was identified as *B. subtilis*was a spore forming gram-positive, rod-shaped, motile, catalase-positive bacterium. Several researchers have also isolated *Bacillus*genus from oil contaminated soils which further validated the result of this study.^32^

**Table 1:** Microscopy and biochemical test on pure isolates

**Samples**	**Gram staining**		**Biochemical Tests**		**Cetrimide**	**Organisms**
		**Oxidase test**	**Catalase test**	**Starch hydrolysis**		
BS1	Purple rods	-ve	+ve	+ve	Creamy rods	*Bacillus subtilis*
PS2	Pink rods	+ve	+ve	-ve	Greenish colonies	*Pseudomonas aeruginosa*
PS5	Pink rods	+ve	+ve	-ve	Creamy colonies	*Citrobacter* spp
BS3	Purple rods	-ve	+ve	+ve	Creamy rods	*Bacillus subtilis*
AW1	Pink rods	+ve	+ve	+ve	Creamy rod	*Escherichia coli*
PS4	Pink rods	+ve	+ve	-ve	Greenish colonies	*Pseudomonas aeruginosa*
PW	Purple rods	-ve	+ve	+ve	Creamy colonies	*Enterobacter*spp
AW2	Purple rods	+ve	+ve	+ve	Creamy rods	*Bacillus*spp

**Key:** BS1, BS3, PS2, PS4 and PS5: Isolates collected from Palm oil Soil sample. AW1-AW2: Isolates from Abattoir sludge (Water). PW: Isolates from palm oil sludge (Water).

Grown on glucose-containing mineral salt medium, the strains PS2 and PS4 produced characteristic diffused green coloured fluorescent pigment containing copious foaming and shiny, large, opaque, serrated-edged colonies. The organisms did not form spores, are gram-negative, rod-like and motile. They were also positive to oxidase and catalase tests but did not produce hydrogen sulphide on MacConkey agar. It was non-lactose fermenting and was identified as *P. aeruginosa*. Isolation of *P. aeruginosa*from palm oil site in this study was not an aberration as *Pseudomonas*genushas a long history of presence in oil-contaminated soil.^33^ However higher presence of *P. aeruginosa*(50%) was found in the soil samples than *B. subtilis*(37.5%). This result is in line with previous research findings which have identified the *Pseudomonas* and *Mycobacterium* genera as very efficient hydrocarbon degrading microorganisms and have been severally employed in bioremediation.^34,35^

### Screening for biosurfactant production

Isolates selection for biosurfactant production was based on the results of their hemolysis, ability to displace and emulsify oil in water-oil mixtures. The bacterial strains, PS2 and BS3 were confirmed biosurfactant producers after displaying significant biosurfactant evaluation results as shown in Table 2 and were therefore selected for further studies. Previous studies^36,37^ demonstrated that hemolytic activity and stabilization of an oil and water emulsion are indicative of biosurfactant production and that it could be used as rapid methods for bacterial screening. In this study some isolates were positive for hemolytic activity. There exists a link between high emulsifying abilities of microorganisms and biosurfactant production.^38^ Emulsification activity is often used as a criterion for determining the potential for biosurfactant production, as it is opined that emulsifying activities (E_24_) determine the productivity of bio-emulsifier.^39^ Emulsification index of ≥30% shows significant good emulsification activity.^40^ Isolates BS1 (50.05%), PS2 (60.68%), BS3 (55.18%) and PS4 (60.77%) have significant emulsification indices above 50% and stand out to be promising biosurfactant producers. Another method of screening for biosurfactant production carried out in this study was oil displacement property. It is a rapid and an easy-to-do test requiring very simple equipment and a small sample volume.^36^ In this current study, 44% of isolates were found to exhibit oil displacement using the oil-spreading method. Thus, the bacterial strains, BS3 and PS2 were confirmed biosurfactant producers since they showed oil displacement of 18.00 and 19.33 mm (Table 2). Earlier reports^41,42^ suggested that areas of well-formed oil displacement were indicative of biosurfactants production. However, the molecular mechanism by which surfactants displace oil is still unclear.^13^

**Table 2:** Biosurfactant production screening result

**Isolates**	**Emulsification index (%)**	**Hemolytic test**	**Oil displacement ability**
BS1	50.05±0.65	β-hemolysis	Yes
PS2	60.68±1.47	β-hemolysis	Yes
BS3	55.18±1.16	α-hemolysis	Yes
PS4	60.77±0.79	α-hemolysis	Yes
PS5	35.18±1.43	α-hemolysis	No
AW1	24.79±3.11	γ-hemolysis	No
PW	35.77±2.02	β-hemolysis	Yes
AW2	24.32±2.03	α-hemolysis	No

**Key:** BS1, BS3, PS2, PS4 and PS5: Isolates collected from Palm oil Soil sample. AW1-2; Isolates from abattoir sludge. PW; Isolates from Palm oil sludge.

### UV irradiation of selected isolates and biochemical characterization

#### Colony forming units of selected isolates after UV irradiation

The colony forming units of isolates obtained were recorded against different times of exposure to UV irradiation. The number of viable colonies reduced in increasing order of exposure time as presented in Table 3.

**Table 3:** Number of bacterial colonies (CFU/mL) (10-^6^ dilution factor) after UV exposure

**Isolates**	**Unexposed (Control)**	**Exposure time (s)**						
		**5**	**15**	**30**	**60**	**300**	**600**	**900**
BS1	1.65 × 10^8^	1.36 × 10^8^	1.11 × 10^8^	5.40×10^7^	1.90×10^7^	1.10×10^7^	None	None
PS2	TNTC	2.05 × 10^8^	1.65× 10^8^	9.50×10^7^	4.60×10^7^	2.10×10^7^	None	None
BS3	2.18 × 10^8^	1.70 × 10^8^	1.30 × 10^8^	8.10×10^7^	2.10×10^7^	1.40×10^7^	None	None
PS4	1.97 × 10^8^	1.49 × 10^8^	1.04 × 10^8^	7.40×10^7^	3.20×10^7^	2.60×10^7^	None	None

All values are expressed as mean, n= 3. BS1: *Bacillus subtilis*mutant from palm oil soil sample. PS2: *Pseudomonas aeruginosa*mutant from palm oil soil sample. BS3: *Bacillus subtilis*mutant from palm oil sludge sample. PS4: *Pseudomonas aeruginosa*mutant from palm oil sludge sample.

#### Biochemical reactions of UV-irradiated Pseudomonas aeruginosa (PS2) and Bacillus subtilis (BS3)

The promising mutants from biochemical reactions of UV irradiated isolates, PS2 and BS3 were considered adequate for fermentation process and consequently used for the rest of the study as they both represent the two isolates of interest *P. aeruginosa*and *B. subtilis*respectively (Table 4). Strain improvement study allows for selection of strains with altered gene regulation and may modulate the ability to produce secondary metabolites.^41^ Researchers have shown that UV, X- and γ-radiation could yield novel bioactive compounds in plants and animal models.^43-45^ In the present study, after exposure of biosurfactant producers to ultraviolet light at different time intervals, various biochemical results indicated that the organisms were mostly not affected by short-term exposure (0-60 seconds) to UV light. Since all biochemical tests gave negative results between 600-900 seconds of isolates exposure to UV rays coupled with confirmatory result by culturing the remains of the isolates on nutrient agar where no growth was recorded, it can be concluded that 600-900 seconds exposure to UV rays destroyed the isolates completely while different growth and result patterns between 15 to 300 seconds of UV rays indicated that the genetic makeup of the isolates were modified leading to various biochemical results recorded.

**Table 4:** Biochemical reactions of UV-irradiated PS2 and BS3

**Isolates**	**Biochemical tests**	**Unexposed (Control)**	**Time of exposure to UV rays (s)**						
			**5**	**15**	**30**	**60**	**300**	**600**	**900**
PS2	Cetrimide	**+**	**+**	**+**	**+**	**+**	**-**	**-**	**-**
	Oxidase	**+**	**+**	**+**	**+**	**+**	**+**	**-**	**-**
	Catalase	**+**	**+**	**+**	**+**	**+**	**+**	**-**	**-**
	Nitrate	**+**	**+**	**+**	**-**	**-**	**-**	**-**	**-**
	Citrate	**+**	**+**	**+**	**+**	**+**	**+**	**-**	**-**
	Indole	**-**	**-**	**-**	**-**	**-**	**-**	**-**	**-**
	Glucose fermentation	**+**	**+**	**+**	**+**	**+**	**+**	**-**	**-**
	Starch hydrolysis	**+**	**+**	**+**	**+**	**+**	**-**	**-**	**-**
BS3	Oxidase	**+**	**+**	**-**	**-**	**-**	**-**	**-**	**-**
	Catalase	**+**	**+**	**+**	**+**	**+**	**+**	**-**	**-**
	Nitrate	**+**	**+**	**+**	**-**	**-**	**-**	**-**	**-**
	Citrate	**+**	**+**	**+**	**+**	**+**	**+**	**-**	**-**
	Indole	**+**	**+**	**+**	**+**	**+**	**+**	**-**	**-**
	Glucose fermentation	**+**	**+**	**+**	**+**	**+**	**+**	**-**	**-**

**Key:** (-): Negative reaction after UV exposure. (+): Positive reaction after UV exposure.

#### Starch hydrolysis test

The ability of the isolates to break down different sugars including glucose, sucrose, lactose and maltose was tested after UV irradiation and the observations recorded as shown in Table 4. Organisms exposed to prolonged period of irradiation showed immediate complete dark coloration while those exposed to UV irradiation between 300-900 seconds of experiment gave a negative starch hydrolysis result.

#### Cetrimide agar test

Isolates PS2 and BS3 were exposed to varying lengths of time of UV light and organisms from each plate grown on Cetrimide agar. The effects of exposure of isolates to UV irradiation indicated that Cetrimide agar test was positive for unexposed organisms (negative control) up till those exposed for 60 seconds, as the plates displayed a greenish colour (growth). A negative Cetrimide agar result was recorded in plates exposed to 300-900 seconds of UV irradiation as shown in Table 4.

#### Growth kinetics and evaluation the production of biosurfactant

**Figure 1:**
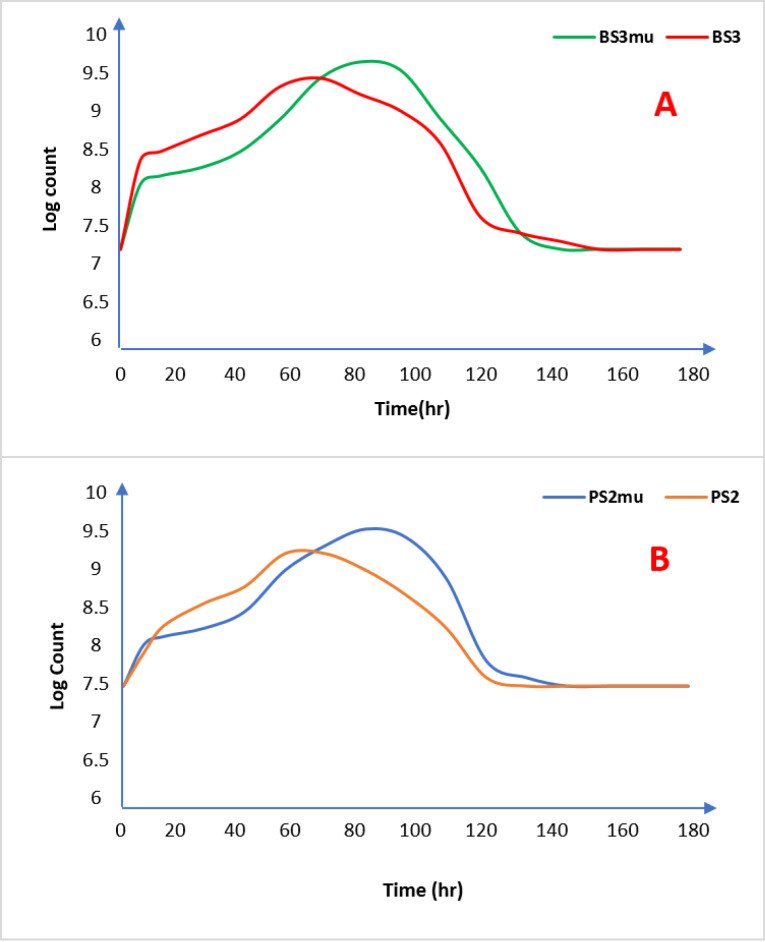
Growth curve of Wild and mutant strains of test organisms. [A =*Bacillus*spp. (BS3) and B =* Pseudomonas* spp. (PS2)] Note: BS3 = *Bacillus*spp-wild strain, BS3mu = *Bacillus*spp-mutant strain. PS2 = *Pseudomonas*spp-wild strain, PS3mu = *Pseudomonas*spp-mutant strain.

The kinetics of growth of both the wild and the mutant strains of *Bacillus*sp and *Pseudomonas*sp are represented in Figure 1a and Figure 1b respectively. The growth curves of the wild strains (BS3 and PS2) when compared with the mutants are similar as mutant strains picked up slower in the fermentation media than wild strains but had higher production output after the 72 hours mark unlike the wild strains that peaked at 60 hours of incubation but with lower biomass production. The bulk of the biosurfactant was produced during the stationary phase of the growth curve as there was no further experimental production after 72 hours by both wild and mutant strains, corroborating previous literatures that the production of biosurfactants is considered as secondary metabolites.^42^

The delay in cell growth by the mutant strains could be connected to the enzymatic manipulation of the isolates brought about by UV mutagenesis as previously observed in biochemical tests of the mutants. Similar to the wild strains (BS3 and PS2), the biomass concentration of the mutants (BS3_mu_ and PS2_mu_) rose instantly after inoculation, signifying appropriate growth conditions in the fermentation media.

The observed cessation of cell growth at 72 hours demonstrates that the culture has reached the stationary phase.^42^ At this growth phase, the concentration of the biosurfactant biosynthesis was recorded as shown in Table 5. The mutant strains produced higher biosurfactants as secondary metabolites than wild strains with BS3mu and PS2mu producing a dry weight biosurfactant of 1112 mg/L and 992 mg/L respectively as against wild strains of BS3 and PS2 that yielded only 905 mg/L and 878 mg/L respectively.

**Table 5:** Assignment of functional groups to piroxicam and solid dispersion

**Functional groups**	**PD**	**PS3**	**PS6**	**BS3**	**BS6**
Secondary Amine N-H Stretch	3391.9	3391.9	3391.9	3391.9	3391.9
Aromatic C-H stretch		3067.6	3067.6	3067.6	3067.6
CH asymmetric stretch of CH_3_		2922.2	2922.2	2922.2	2922.2
CH symmetric stretch of CH_2_		2858.9	2858.9	2858.9	2858.9
CH stretch of CH_2_			2109.7	2109.7	2113.4
C=O stretch of ester carbonyl		1736.9	1736.9	1736.9	1736.9
C=O stretch of secondary amide	1636.9	1636.3	1636.9	1636.9	1636.9
C=C, C=N ring stretch	1528.2	1524.5	1524.5	1524.5	1524.5
asymmetric C-H bend of CH_3_	1435	1435.0	1435.0	1435.0	1435.0
CH stretch of methyl group	1353	1349.3	1349.3	1349.3	1349.3
C=O stretch of CH_2_	1274.7	1274.7	1274.7	1274.7	1274.7
Symmetric S(=O)_2_ stretches	1151.7	1177.8	1177.8	1177.8	1177.8
Symmetric S(=O)_2_ stretches		1140.6	1140.6	1140.6	1140.6
C-O Stretch	1032.5	1032.5	1032.5	1032.5	1032.5

### Properties of biosurfactants produced by UV irradiated isolates

#### Emulsification index

**Figure 2:**
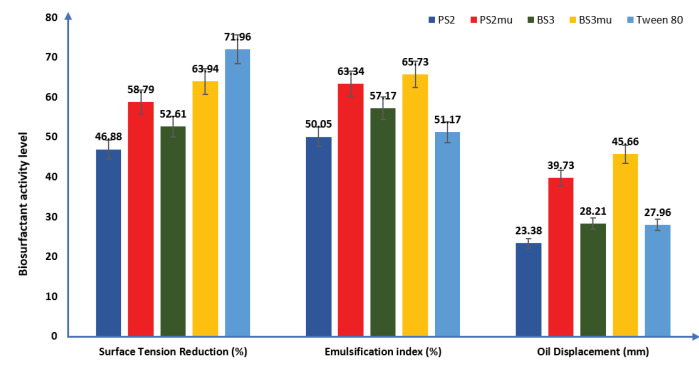
Evaluation of biosurfactants properties: (PS2 = Wild *Pseudomonas* Strain, PS2mu = Mutant *Pseudomonas* strain; BS3 = Wild *Bacillus* Strain, BSmu = Mutant *Bacillus* Strain). Data were presented as mean ± standard error of mean (SEM).

Emulsification indices of the biosurfactants are shown in Figure 2. The order of emulsification of the biosurfactants (2 mL in 3 ml of vegetable oil) is as follows: BS3_mu_ (65.73%) > PS2_mu_ (63.34%) > BS3 (57.17%) >PS2 (50.05%). The produced biosurfactant from mutant strains had better emulsification index than the commercially available surfactant used in this study with an emulsification index of 51.17%.

#### Oil displacement

The ability of biosurfactants to spread vegetable oil in millimetre (mm) is presented in Figure 2. The results showed that mutant strain of BS3 had the highest oil displacement activity and was able to spread the oil at the middle of Petri dish used by 45.66 mm, this was followed by biosurfactant produced by mutant PS2 (39.73 mm), while wild strain of PS2 had the lowest but significant oil displacement activity, spreading the vegetable oil by 23.38 mm as against 28.21 mm oil displacement by wild strain of BS3 when measured from the centre of the used Petri dish.

#### Surface tension

The result of the ability of the produced biosurfactants to reduce surface tension is presented in Figure 2. The mutant strain of BS3_mu_ had the highest activity as it lowered the tension of the surface film of distilled water from 72 D/cm to 25.96 D/cm, followed by PS2_mu_ with a reduction in surface tension to 29.67 D/cm. The wild strain of PS2 had the least surface tension reduction ability (from 72 to 38.25 D/cm) while the wild strain of BS3 reduced the surface tension of distilled water from 72 to 34.12 D/cm. However, Tween 80 used as commercially available surfactant demonstrated a higher surface tension reduction ability under same atmospheric conditions than the generated biosurfactants by reducing the surface tension of distilled water to 20.19 D/cm.

The biosurfactants produced by mutant strain of the *Bacillus*spp (BS3_mu_)was able to reduce the surface tension of distilled water lower than that of *Pseudomonas*spp (PS2_mu_). This in addition to the higher emulsification index and oil displacement ability demonstrated by the former strain indicates that BS3 is a better biosurfactant producer than PS2. This discovery is in agreement with reported findings.^46,47^ It was also reported in other previous works^3,19,48^ that a good surface acting agent can reduce the tension of the surface film of water by ≤50%.

Dry weight (g/L) of the biosurfactant produced by wild and mutant strains is shown in the Supplementary Table 1.

#### Critical micelle concentration

**Figure 3:**
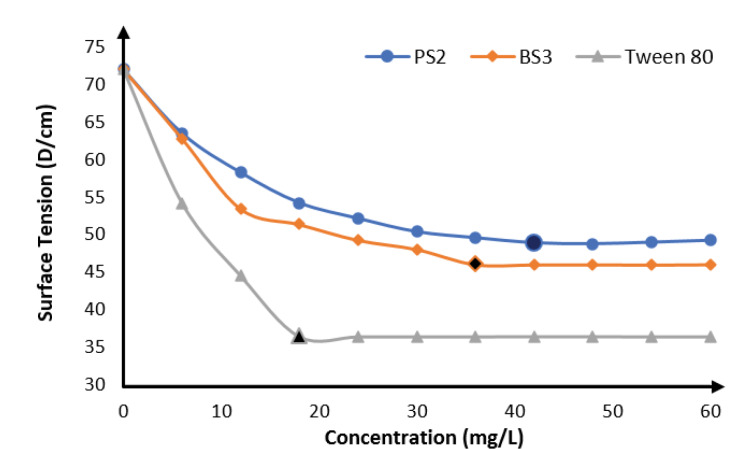
Critical micelle concentrations of biosurfactants produced by UV irradiated *Pseudomonas aeruginosa*(PS2) and *Bacillus subtilis*(BS3) relative to Tween 80.

The results of CMC determination are shown in Figure 3 with biosurfactants produced by mutant BS3 having the lowest, (36.0 mg/L) while that produced by mutant PS2 had a CMC of 42.0 mg/L. The commercially available surfactant used in this study gave a lower CMC of 18 mg/L than the produced biosurfactants. This is related to the higher surface tension ability earlier displayed by the commercial surfactant.

It can also be interpreted from the result that biosurfactants produced by BS3 having a lower CMC than PS2 will therefore be a more potent biosurfactant in surface tension reduction than biosurfactants produced by PS2 because lower quantity will be required. Similar result was obtained by some other researchers where *Pseudomonas*strain was used.^49,50^ *P. aeruginosa*strains had been reported to produce biosurfactants capable of lowering the tension at surface film of distilled water from 72 to 30 D/cm and with CMC ranges of 5–200 mg/L.^51^ The different CMC values of *Pseudomonas* biosurfactants had been attributed to differences in molecular structures, unsaturated bonds, branching as well as aliphatic chains lengths of rhamnolipids.^52^ Biosurfactants with low CMC and high surface activities are easily biodegradable and so are eco-friendly and are amenable to environmental applications.^50^

#### Antimicrobial activity

From the *in vitro* antibacterial and antifungal screening of the extracted biosurfactants, the produced biosurfactants did not show positive activity against either the gram-positive or gram-negative bacterial strains (see Supplementary Table 2 of the Supplementary file). The negative antimicrobial activities may be partly due to low concentrations of biosurfactants used, this result is in contrast with earlier reports on the antimicrobial actions of the biosurfactants from *Pseudomonas* and *Bacillus*.^53,54^ Moreover, the low concentrations of the produced biosurfactants may be attributed to the extraction process adopted in this study which involved the use of millipore membrane filter rather than the conventional chemical method recorded in literature.^22,47^ Previous studies where higher concentrations of biosurfactants viz-a-viz significant positive antimicrobial results were obtained used different chemical combinations for the extraction process which partly made it possible for complete extraction of active ingredients from the production cells. That was not the case in this study as the research attempted for the complete elimination of chemical reagents interference with the activities of the produced biosurfactants; as these chemicals may confer toxicity on the generated biosurfactants.

#### Wavelength of maximum absorption and calibration curve for piroxicam

The UV spectrum of piroxicam was evaluated using Agilent Cary 60 UV-Vis Spectrophotometer (Santa Clara US). The peak absorption at 358 nm was chosen in preference to 205 nm because the latter may easily give unstable reading during subsequent analyses since it is very close to x-ray regions and impurities may also give a similar peak at that wavelength region.^55^ This value was maintained in subsequent UV determinations during dissolution and solubility studies. The linear regression of piroxicam concentration against its absorbance in distilled water is y=0.007x-0.0303, with r^2^=0.9946.

### Characterization of piroxicam-biosurfactants solid dispersions

#### FTIR spectra of piroxicam and piroxicam-biosurfactant solid dispersions

In order to identify functional groups, present in the generated biosurfactants, FTIR was conducted. FTIR is proven as a useful analysis for the identification of chemical bonds and functional groups occurring in bioactive fraction of an unknown biosurfactant and enables prediction of its chemical nature.^56^

**Figure 4:**
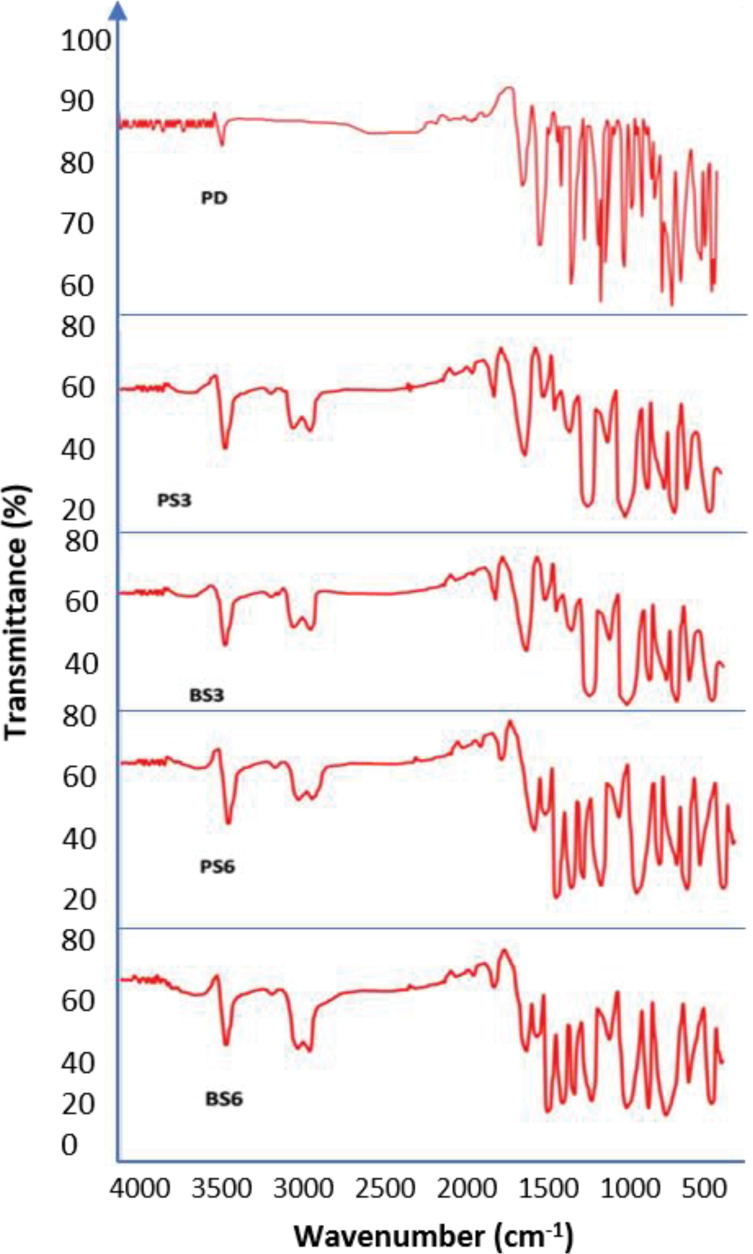
FTIR spectra. (PD =Pure Piroxicam; PS3 =3ml of piroxicam and biosurfactant produced by *Pseudomonas aeruginosa*; BS3 =3ml of piroxicam and biosurfactant produced by *Bacillus subtilis*; PS6 =6 mL of piroxicam and biosurfactant produced by* Pseudomonas aeruginosa*; BS6 = 6ml of piroxicam and biosurfactant produced by* Bacillus subtilis*).

FTIR analysis identifies the functional groups present in the generated biosurfactants. The emission spectra of the piroxicam-biosurfactant complexes as obtained using Agilent Cary 60 UV-Vis Spectrophotometer (Santa Clara US) in the range of 650–4000/cm with a resolution of 1/cm were shown in Figure 4. Table 5 gives the assignment of the functional groups present in the samples. All the functional groups present in pure piroxicam spectrum were retained in the solid dispersion. This indicates that there was no chemical interaction between the piroxicam and the biosurfactants. The FTIR spectra obtained from the biosurfactants generated from the two mutant strains of *P. aeruginosa*(PS2) and *B. subtilis* (BS3) when compared with previous reports,^56,57^ show that various secondary metabolites generated contained glycolipids and lipopeptide. This indicates that the mutant strains were able to produce more than one type of biosurfactant as compared to single pure strains earlier reported.^58^

The various chemical bonds recorded in the biosurfactant-piroxicam complexes that are not in the pure piroxicam drug as shown in Table 5 indicates that the different BS-piroxicam complexes contained additional chemical compounds to the compounds originally present in the piroxicam drug.

#### Differential scanning calorimetry

**Figure 5:**
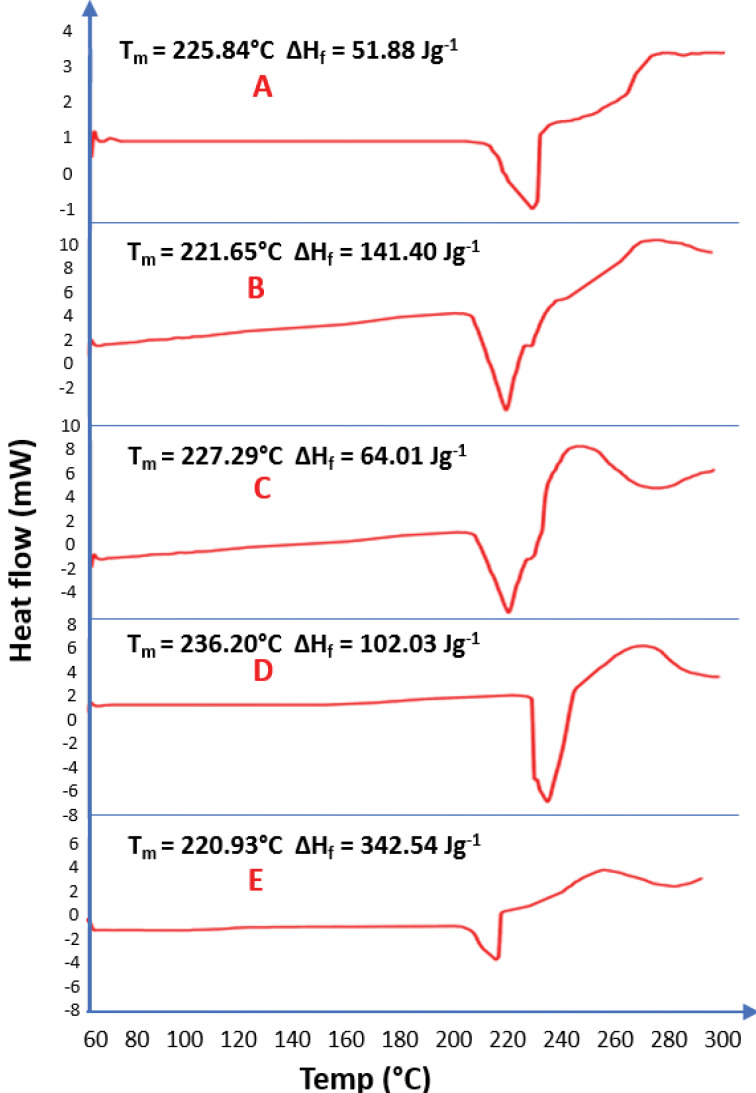
DSC results (A=Pure Piroxicam; B= Solid dispersion of 6ml of biosurfactant produced by *Pseudomonas aeruginosa* and piroxicam; C = Solid dispersion of 3ml of biosurfactant produced by *Pseudomonas aeruginosa* and piroxicam; D= Solid dispersion of 3ml of biosurfactant produced by *Bacillus subtilis* and piroxicam; E= Solid dispersion of 6ml of biosurfactant produced by *Bacillus subtilis* and piroxicam)

DSC thermo-grams of pure piroxicam and biosurfactants-piroxicam solid dispersions recorded using the Mettler Toledo DSC 822e (Columbus, Ohio, USA) are shown in Figure 5.

DSC thermograms of piroxicam and biosurfactants-piroxicam complexes showed that the thermograms are similar. The pure piroxicam drug has a melting point of 236.20°C considerably higher than the melting points of 3 mL of biosurfactant-piroxicam solid dispersion from BS3 (225.84°C) and PS2 (227.29°C) and 6 ml of the same complex produced from BS3 (221.65°C) and PS2 (220.93°C). This clearly showed that the higher the biosurfactant concentration in the BS-Piroxicam complex, the lower the melting points and the higher the percentage of solubility.^59^ The heat of fusion recorded in this study gave a clear indication that higher thermal energy (102.03 J/g) is needed to dissolve a unit mass of pure piroxicam (PD) as compared to an equal mass of 3 ml biosurfactant-piroxicam complex produced by PS2 (64.01 J/g) and BS3 (51.88 J/g). This result was however challenged as increase in biosurfactant concentration was expected to further lower the heat of fusion since biosurfactant would reduce the intermolecular force of the drug particles due to surface tension reduction ability, but higher concentrations of biosurfactant resulted in higher heats of fusion; 6 mL of both BS3 (141.40 J/g) and PS2 (342.54 J/g) biosurfactant-piroxicam complexes resulted in a greater heat of fusion than observed in both pure drug sample and 3ml of biosurfactant-piroxicam dispersion. This could be due to higher mass of the dried 6ml of piroxicam-solid dispersion (6.200 mg) of the BS3 and PS2, compared to about 4.200 mg of pure drug and 3 mL of the complexes used during analysis as it is expected that higher mass of substance will require more heat energy for complete dissolution of solid particles of the drug.^60^

#### X-ray powder diffraction

**Figure 6:**
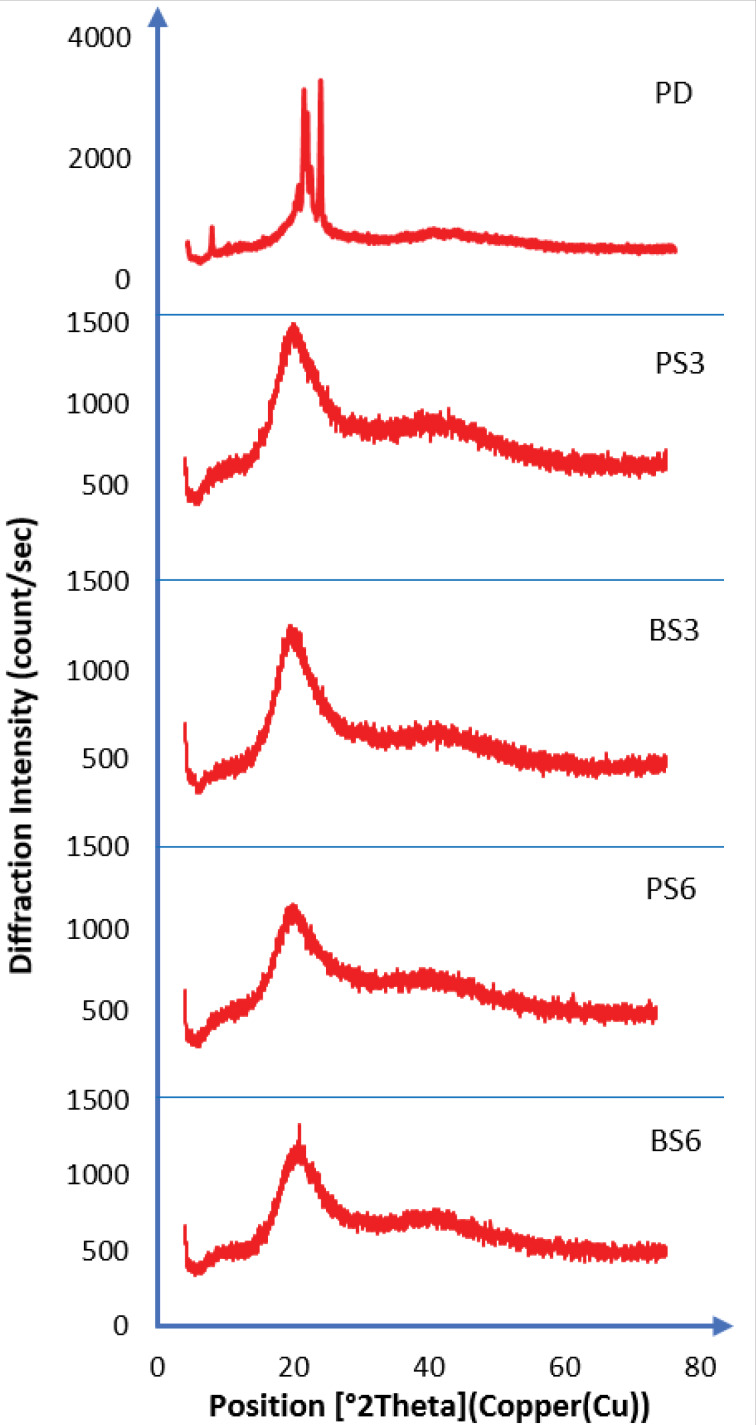
XRPD results (PD=Pure Piroxicam; PS3= Solid dispersion of 3 ml of biosurfactant produced by *Pseudomonas aeruginosa* and piroxicam; PS6 = Solid dispersion of 6 ml of biosurfactant produced by *Pseudomonas aeruginosa* and piroxicam; BS3= Solid dispersion of 3 ml of biosurfactant produced by *Bacillus subtilis* and piroxicam; BS6= Solid dispersion of 6 ml of biosurfactant produced by *Bacillus subtilis* and piroxicam).

XRPD was used to qualitatively characterize the crystalline nature of the biosurfactants. The diffractograms are recorded in Figure 6. FTIR and DSC results indicated the non-crystallinity of the biosurfactants, which was confirmed by x-ray diffractometer results. The X-ray diffraction pattern of piroxicam exhibited sharp, intense and concentrated peaks showing the crystalline nature of piroxicam. It showed maximum peak intensities of the piroxicam drug at 2*θ* of around 20.98°, PS3 (21.00°), BS3 (23.39°), PS6 (23.42°), and BS6 (23.45°). The intensity of the diffraction peaks observed in pure piroxicam indicated that the particles were fairly well-crystallized. The crystallinity of the particles in both 3 and 6 mL of biosurfactant-piroxicam complexes disappeared as ratio of the generated biosurfactant increased. The XRPD data confirmed that the biosurfactant has unique crystallinity, and observations from biosurfactant-piroxicam solid dispersion showed that the various biosurfactants successfully amorphized the pure drug, leading to a disruption of the regular molecular structure of the piroxicam. Consequently, the melting points of the biosurfactant-piroxicam complexes were significantly lowered leading to reduced heat of fusion, higher solubility and dissolution in biosurfactant complexes. This result supports the findings from both FTIR spectra and DSC. The XRD peak intensity of the prepared biosurfactant-piroxicam solid dispersions was lower and less defined in comparison to that produced by the pure piroxicam drug.

#### Solubility

**Figure 7:**
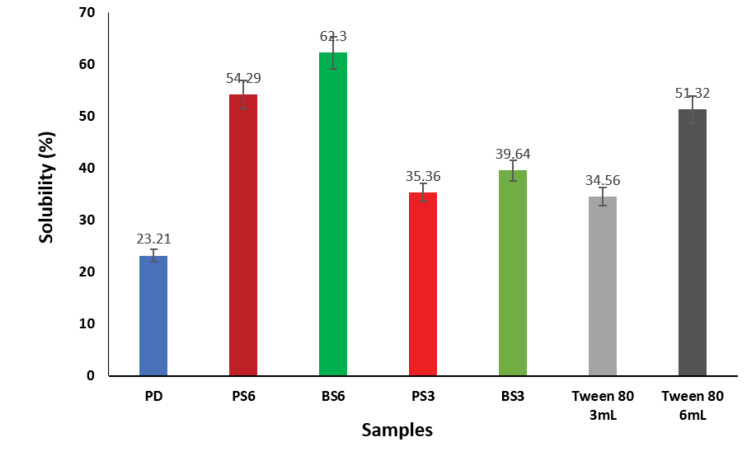
Effects of biosurfactants produced by *Pseudomonas aeruginosa*and* Bacillus subtilis*on solubility of piroxicam.
**PD**: Pure Piroxicam; **PS3**: Solid dispersion of 3 mL of biosurfactant produced by *P. aeruginosa*and piroxicam; **BS3**: Solid dispersion of 3 mL of biosurfactant produced by *B. subtilis* and piroxicam; **PS6**: Solid dispersion of 6 mL of biosurfactant produced by* P. aeruginosa* and piroxicam; **BS6**: Solid dispersion of 6 ml of biosurfactant produced by* B. subtilis* and piroxicam.

The solubility of a drug affects its bioavailability because a drug with poor solubility profile cannot be easily absorbed through the membranes and thus its permeability will be low.^61^ In the present study, the solubility of pure piroxicam and piroxicam-biosurfactant solid dispersions was conducted at 37°C in distilled water. The results of solubility studies are shown in Figure 7. It was observed that 6 mL of BS (BS6) gave the highest solubility of 62.3% of the biosurfactant-piroxicam solid dispersions after 48 hours. A 3 mL volume of the same sample (BS3) showed a reduced solubility of 39.64%. Addition of 6 ml of PS (PS6) biosurfactant to piroxicam produced 54.29% solubility when compared to 35.36% recorded when 3 ml of the same biosurfactant (BS3) was used. The pure drug sample gave the lowest solubility of 23.21% within the same period while the commercial surfactant used (Tween 80) resulted in 51.32% solubility. The solubility studies revealed that the two produced biosurfactants (BS3 and PS2) had fairly good solubility profile (>10 μg/mL). Increase in solubility of biosurfactant-piroxicam complex may be due to improved wetting of drug or reduction in crystallinity of the drug as shown via the DSC and XRPD results. Oral drugs having a minimum solubility of 10 μg/mL and log*P*≥2 have optimal absorption indices.^62^ Drug dissolution is a function of its solubility and a drug product with aqueous solubility < 0.1 mg/mL often have limited absorption due to dissolution problems.^63^

#### Dissolution profile

**Figure 8:**
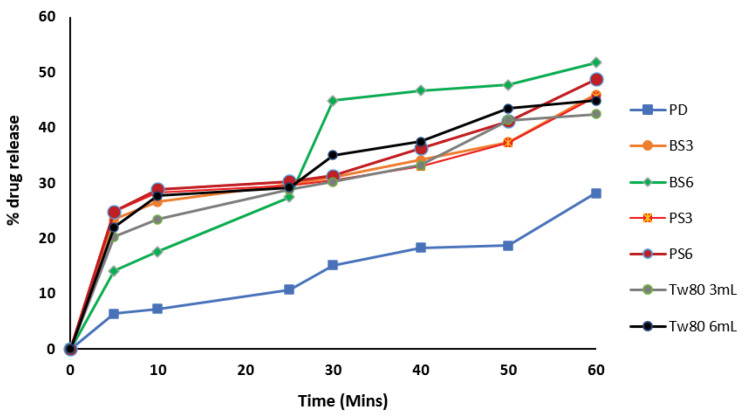
Dissolution profile of piroxicam-biosurfactant solid dispersion.
**PD:** Pure Piroxicam; **PS3:** Solid dispersion of 3 mL of biosurfactant produced by *P. aeruginosa*and piroxicam; **BS3**: Solid dispersion of 3 mL of biosurfactant produced by *B. subtilis* and piroxicam, **PS6:** Solid dispersion of 6 mL of biosurfactant produced by* P. aeruginosa* and piroxicam, **BS6:** Solid dispersion of 6 ml of biosurfactant produced by* B. subtilis* and piroxicam

Results of dissolution study as shown in Figure 8 showed that 6 ml of BS (BS6) gave the highest dissolution of 51.71% after a period of 60 min in the dissolution medium while 3 ml of the same sample (BS3) gave 46.00% dissolution. 6 ml of PS (PS6) in the biosurfactant-piroxicam solid dispersion gave 48.71% dissolution as compared to 45.57% dissolution observed with 3 mL of PS (PS3). The pure piroxicam drug sample (PD) showed the least dissolution of 28.14% at the end of 60 minutes dissolution study while 6ml of Tween 80 which was used as positive control gave 44.89% solubility at 60 minutes.

*In vitro*dissolution profiles of all piroxicam-biosurfactant solid dispersions were better than that of pure piroxicam and commercially available sample. The drug release percentage increased as the concentration and time of treatment increased in the pure piroxicam drug, Tween 80 and biosurfactants. These results confirmed earlier results in this study where biosurfactants produced by *B. subtilis* (PS3) gave a lower CMC and higher surface activity than those produced by *P. aeruginosa*(PS2).

The improved solubility and dissolution of piroxicam by the generated biosurfactants is an indication that these biosurfactants will be able to solve bioavailability problems of poorly water-soluble drugs. This position was earlier established in literature.^64^ Improved oral absorption of drugs is observed when administered with surface-active agents due to the improved drug solubility and better dissolution rates because of the wetting effects of surfactants generally.It had been previously proven that at high surfactant concentrations, the dissolution medium is more viscous. Studies^65,66^ showed that the presence of surfactants in the dissolution medium enhances drug products wettability and dissolution rate, even at surfactant concentrations lower than the CMC.

## Conclusion

The ultraviolet irradiation treatment of the test *P. aeruginosa*(PS2)and *B. subtilis* (BS3) isolates produced mutant strains that have special features than the wild strainswhich have been previously used in production of biosurfactants. The mutant strains were able to survive adverse environmental conditions and produced useful biosurfactants which demonstrated exceptional pharmaceutical activities as there were improved piroxicam solubility and dissolution, surface tension reduction ability and high emulsification activity. In addition to biosurfactant production with high activity, the study revealed a cheaper process of developing biosurfactants using industrial wastes thereby achieving a double benefit of reducing environmental pollution and producing valuable biotechnological products (biosurfactants). This study generated biosurfactants which are suitable alternatives to the widely used chemical surfactants in pharmaceutical industries.

## Ethical Issues

Not applicable.

## Conflicts of Interest

The authors declare no conflict of interest.

## Supplementary Materials

Supplementary file 1 contains Tables S1 and S2.

## Acknowledgments

The authors are thankful to Mr. Emmanuel Okwor of National Agency for Food and Drug Administration and Control (NAFDAC), Zonal Laboratory; Agulu, Mrs. Chinwe of Pauco Pharmaceuticals, Awka and to Mr. Issa Abdulahi of Chemical Engineering Department, Ahmadu Bello University, Zaria for their immense contributions to the success of this research.
