# Supplementary material for: Biosurfactant Production Using Mutant Strains of Pseudomonas aeruginosa and Bacillus subtilis from Agro-industrial Wastes
Source: Adv Pharm Bull. 2020 Oct 20;11(3):543–56. doi: 10.34172/apb.2021.063 (PMC8421616; doi:10.34172/apb.2021.063)
Supplement: Supplementary file 1 — contains Tables S1 and S2. [file apb-11-543-s001.pdf]

Biosurfactant Production Using Mutant Strains of *Pseudomonas aeruginosa* and *Bacillus subtilis* from Agro-industrial Wastes

Supplementary Table 1. Evaluation of biosurfactants produced by wild and mutant strains

Sample	Surface Tension (D/cm)	Emulsification Index (%)	Oil Displacement (mm)	Dry weight of biosurfactants (g/L)	P-value
**PS2**	38.25±0.32	50.05±1.03	23.38±0.98	0.878±0.61	0.0*
**PS2mu**	29.67±0.44	63.34±1.47	39.73±2.03	0.992±0.01	0.0*
**BS3**	34.12±1.12	57.17±0.32	28.21±1.21	0.905±1.04	0.0*
**BS3mu**	25.96±0.38	65.73±0.65	45.66±0.58	1.112±0.11	0.0*
**Tween 80**	20.19±1.18	51.17±2.03	27.96±1.20	NA	0.0*

All data were statistically described in terms of mean and standard error (±S. E); *, represent highly significant difference (P<0.001)

Supplementary Table 2. Antimicrobial activity of the biosurfactants

**Isolates**		**Inhibition zone diameter (mm) at different concentrations (mg/L)**							
		**0**	**10.2**	**20.4**	**30.6**	**61.2**	**122.4**	**PC**	**NC**
**Bacteria**	*Escherichia coli*	N	N	N	N	8	11	25	N
	Salmonella	N	N	N	N	6	14	20	N
	Shigellae spp	N	N	N	N	N	N	17	N
	Listeria spp	N	N	N	N	N	N	21	N
	Vibrio spp	N	N	N	N	N	N	18	N
	Staphylococcus aureus	N	N	N	N	N	N	22	N
**Fungi**	*Aspergillus niger*	N	N	N	N	N	N	31	N
	Aspergillus flavus	N	N	N	N	N	N	36	N
	Candida albicans	N	N	N	N	N	N	34	N

Diameters are recorded as mean values of triplicate measurements

KEY:
N: No zone of inhibition
Positive Control (PC): Gentamicin (10 μg) for Bacteria; Clotrimazole (50 μg) for Fungi
Negative Control (NC): Dimethyl Sulfoxide (DMSO) (5 mm) for both bacteria and fungi

Supplementary
